# Gemcitabine Plus Cisplatin Versus Non-Gemcitabine and Cisplatin Regimens as Neoadjuvant Treatment for Cholangiocarcinoma Patients Prior to Liver Transplantation: An Institution Experience

**DOI:** 10.3389/fonc.2022.908687

**Published:** 2022-06-02

**Authors:** Maen Abdelrahim, Abdullah Esmail, Jiaqiong Xu, Godsfavour Umoru, Hadeel Al-Rawi, Ashish Saharia, Ala Abudayyeh, David Victor, Robert McMillan, Sudha Kodali, Rafik M. Ghobrial

**Affiliations:** ^1^ Section of GI Oncology Department of Medical Oncology Houston Methodist Cancer Center, Houston, TX, United States; ^2^ Cockrell Center of Advanced Therapeutics Phase I program, Houston Methodist Research Institute, Houston, TX, United States; ^3^ Department of Internal Medicine, Weill Cornell Medical College, New York, NY, United States; ^4^ Houston Methodist Cancer Center, Houston Methodist Hospital, Houston, TX, United States; ^5^ Center for Outcomes Research, Houston Methodist Research Institute, Houston, TX, United States; ^6^ Department of Pharmacy, Houston Methodist Hospital, Houston, TX, United States; ^7^ Houston Methodist Hospital, JC Walter Jr Center for Transplantation and Sherrie and Alan Conover Center for Liver Disease and Transplantation, Houston, TX, United States; ^8^ Section of Nephrology, Division of Internal Medicine, The University of Texas MD Anderson Cancer Center, Houston, TX, United States

**Keywords:** cholangiocarcinoma, hepatocellular carcinoma, gemcitabine, cisplatin, immunotherapy, liver transplantation, transplant oncology, and FOLFIRI

## Abstract

**Background:**

Cholangiocarcinoma management is constantly being updated in view of existing evidence in order to establish practice guidelines and consensus statements. However, the available treatment guidelines to optimize outcomes for cholangiocarcinoma patients who require liver transplantation are still controversial. This study contributing to the cholangiocarcinoma care field by investigating a new promising neoadjuvant therapy that might be help to grant the liver transplant option to the patients with cholangiocarcinoma. Here, we evaluate and compare the potential efficacy of chemotherapy combination of Gemcitabine plus Cisplatin versus non- Gemcitabine and Cisplatin regimens as a neo-adjuvant treatment for cholangiocarcinoma patients prior to liver transplantation.

**Methods:**

In this retrospective study, patients with locally advanced, unresectable, hilar, or intrahepatic cholangiocarcinoma with no evidence of extrahepatic disease or vascular involvement were treated with either the combination of neo-adjuvant Gemcitabine plus Cisplatin with no radiation or other standard options of neo-adjuvant treatment. All patients included received chemotherapy prior to being listed for liver transplantation at a single cancer center in collaboration with the same institution’s transplant center according to an open-labeled, and centers-approved clinical management protocol. Patients were listed for liver transplantation if they had a minimum of six months of scans showing response or confirmation of disease stability. The primary endpoints were the overall survival and recurrence-free survival after liver transplantation. This report, which was censored on March 18, 2022.

**Results:**

Out of a total of 707 liver transplant recipients were screened, 37 patients were confirmed with a diagnosis of cholangiocarcinoma and only 18 patients (11 males and 7 females) with a median age of 61.83 [interquartile range: 58.27-68.74] met inclusion criteria. Of the 18 patients enrolled, 10 received Gemcitabine/Cisplatin, while 8 patients received either Gemcitabine monotherapy or Capecitabine or FOLFIRI. Months for recurrence after transplantation was 20.1 (IRQ: 20.1-20.1) in the Gemcitabine/Cisplatin group and 9.5 (8.9-12.47) months in the non-Gemcitabine/Cisplatin group (p-value=0.18). Median months of follow-up in the Gemcitabine/Cisplatin group was 28.35 (27.1-32.23) months versus 40.12 (20.6-56.22) months in the non-Gemcitabine/Cisplatin group (p-value=0.33). In non-Gemcitabine/Cisplatin patients, overall survival was 75% (95% CI 31-93%) at both years 1 and 2; 63% (95% CI 23-86%) at years 3 to 5. In Gemcitabine/Cisplatin patients, overall survival was 100% (95% CI 100-100%) at both years 1 and 2; 75% (95% CI 13-96%) at years 3 to 5. Three non-Gemcitabine/Cisplatin patients died at 328 days, 340 days, and 896 days, respectively. One Gemcitabine/Cisplatin patient died at 885 days.

**Conclusion:**

Our findings suggest improved overall survival outcomes with Gemcitabine plus Cisplatin as neo-adjuvant treatment with no concomitant radiation compared to non-Gemcitabine/Cisplatin regimens in patients with cholangiocarcinoma prior to liver transplantation.

## Introduction

Cholangiocarcinoma is classified according to its anatomic site in the biliary tree: distally, peri-hilar, or intrahepatic. Cholangiocarcinoma can occur in a variety of anatomic regions, each of which corresponds to a particular etiology (1, 2). Extrahepatic cholangiocarcinoma (ECCA) is thought to be caused by stem cells in the biliary glands, whereas intrahepatic cholangiocarcinoma (IHCCA) is caused by hepatocyte stem cells (1, 2).

IHCCA is an uncommon malignancy, with only around 8000 cases reported each year in the United States, and accounts for only 3% of all gastrointestinal malignancies diagnosed each year worldwide (3, 4). IHCC has been more common in recent decades, with some studies showing a 14 percent annual increase in frequency since the early 1990s (5). The increased global prevalence of hepatitis C infection, as well as obesity-related non-alcoholic fatty liver disease and non-alcoholic steatohepatitis, which are established risk factors for ICC (5–7), are likely to be contributing to the rise in ICC incidence.

The preferred first-line chemotherapy regimen for advanced cholangiocarcinoma is gemcitabine and cisplatin-based on the randomized, controlled, phase III ABC-02 study (8). This regimen improved OS and PFS by 30% compared to gemcitabine alone. Even so, the median OS was only 11.7 months for the combination and 8.1 months for gemcitabine alone. Other treatments are either gemcitabine or fluoropyrimidine-based (9, 10). For example, gemcitabine may also be combined with oxaliplatin, albumin-bound paclitaxel, and cetuximab, while fluoropyrimidine-based treatments also include cisplatin or oxaliplatin. Interestingly, in a phase II trial with gemcitabine, cisplatin, and albumin-bound paclitaxel, the tumors in 20% of patients who previously had unresectable disease became resectable (11). Overall, various phase II studies have led to category 2A recommendations for gemcitabine with oxaliplatin or capecitabine, capecitabine with oxaliplatin, and the single-agents fluorouracil, capecitabine, and gemcitabine.

There is no specific recommended second-line treatment for CCA. Treatment with FOLFOX (in patients who had previously received cisplatin and gemcitabine) was then validated with the randomized phase III ABC-06 that showed improved OS compared to active symptom control alone (12). Yet so far, with a systematic review of 23 studies including 14 phase II clinical trials, there is insufficient data to recommend any chemotherapy over the others based on efficacy.

For patients with IHCCA, a prognostic scoring system created by researchers at the University of California, Los Angeles has been utilized to improve outcomes over the past 20 years (13, 14). Fluorouracil- or capecitabine-based regimens in conjunction with oxaliplatin, leucovorin calcium, and gemcitabine hydrochloride, are recommended treatment options in the neoadjuvant/adjuvant setting. The first multi-center cooperation sites to describe a prospective case series of protocolized neoadjuvant chemotherapy followed by liver transplantation for patients with IHCCA were Houston Methodist J.C. Walter Jr. Liver Transplant Center and MD Anderson Cancer Center (15). The median cumulative tumor diameter in this study was 14.2 cm, and there was no specified tumor size cut-off. Patients were examined and listed for transplant if tumor radiographic stability was maintained for more than 6 months in this study. The six patients studied had an overall survival rate of 83.3 percent after five years, with a recurrence-free survival rate of 50 percent (16). Furthermore, the International Liver Cancer Association has advised prospective clinical studies of neoadjuvant chemotherapy with liver transplantation (LT) in patients with IHCCA (17). In most centers across the globe, the presence of IHCCA in a cirrhotic liver constituted a contraindication for liver transplantation. However, recent research has indicated that early stage IHCCA may have acceptable outcomes following liver transplantation. In addition, results of another study (18), suggested that 1-year, 3-year, and 5-year actuarial survival rates following LT were 79 percent, 50 percent, and 45 percent, respectively, in patients with late stage disease (single tumor >2 cm or multifocal disease). Consequently, patients with cirrhosis and early stage IHCCA may be candidates for liver transplantation, according to these data.

With regards to management of hilar cholangiocarcinoma (HCCA), more prospective multicenter clinical studies are needed. Patients with unresectable HCCA have been shown to benefit from LT. Also, neoadjuvant chemoradiation treatment prior to LT in patients with unresectable HCCA resulted in a 5-year recurrence-free survival rate of 65 percent (19). To treat unresectable HCCA, several transplant centers in the United States adopt Mayo Clinic criteria which include utilization of chemoradiation followed by LT. Select individuals with unresectable cholangiocarcinoma without intrahepatic or extrahepatic metastases are treated with irradiation and bolus fluorouracil (5-FU), followed by iridium brachytherapy and concurrent extended venous infusion of 5-FU, according to the Mayo protocol. After that, maintenance chemotherapy (i.e., oral capecitabine ambulatory infusion 5-FU) is given until the liver is transplanted. With a survival rate of 60.4 percent, this criterion has significantly improved outcomes. However, given the mixed results of other studies who have utilized this protocol outside of the United States, the resectability vs. unresectability criteria is still debatable (20). Moreover, there is limited data on which specific neoadjuvant chemotherapy regimens are being utilized prior to liver transplantation by centers in the United States who are actively transplanting these patients. Liver transplantation as treatment option for patients with cholangiocarcinoma or hepatocellular carcinoma has been actively evolving in last decade with promising outcomes (21–24). Liver transplant surgery carries a risk of significant complications. There are risks associated with the procedure itself as well as with the drugs necessary to prevent rejection of the donor liver after the transplant such as arterial and venous thrombosis and stenosis, biliary disorders, fluid collections, neoplasms, and graft rejection. In this study, we report and compare overall survival data associated with utilizing combination of Gemcitabine plus Cisplatin versus non- Gemcitabine and Cisplatin regimens as neo-adjuvant treatment with no concomitant radiation for cholangiocarcinoma patients prior to liver transplantation.

## Methods

### Participants

Between August 2008 and March 2022, all patients who got a liver transplant were screened for eligibility. The United Network for Organ Sharing received reports from patients who had either a main or secondary diagnosis. The radiographic assessment and evidence of pathological findings that verified the diagnosis were used to make the CCA diagnosis. The patients were split into two groups: those who received Gemcitabine plus Cisplatin and those who received chemotherapy other than Gemcitabine and Cisplatin. Multi-organ transplants, patients who had not had at least one cycle of treatment prior to transplant, and patients who had received locoregional therapy, such as microwave ablation, radiofrequency ablation, radioembolization, or yttrium-90, were also excluded from the study. Regarding to our liver transplantation protocol at our institution, locally advanced intrahepatic or hilar cholangiocarcinoma was defined as a solitary tumor if greater than 2 cm in diameter or if the multifocal disease was confined to the liver without radiological evidence of extrahepatic, macrovascular, or lymph node involvement. Patients were selected if there was tumor regression on neoadjuvant therapy or if they had shown 6 months of disease stability. Regrading to the decision-making, a multidisciplinary case by case basis were reviewed by the tumor board to choose potentially appropriate candidates composed of medical GI oncologists, transplant surgeons, radiation oncologists, hepatologists, pathologists, and interventional radiologists. Patients who matched the eligibility criteria were then divided into groups according to whether or not they had received at least one cycle of available options drug such as Gemcitabine plus Cisplatin prior to LT.

### Neoadjuvant Therapy

For neoadjuvant treatment, all patients followed standard guidelines. The computed tomography (CT) and magnetic resonance imaging (MRI) images were evaluated by specialized liver radiologists for tumor size and features. Systemic therapy regimens were determined by GI oncologists, a multidisciplinary team of GI oncologists, transplant surgeons, radiologists, and hepatologists assessed each patient.

### Follow-up

Patients underwent a complete blood cell count and prothrombin time test every month, as well as liver function tests for alanine aminotransferase (ALT), aspartate aminotransferase (AST), bilirubin, albumin, and Carcinoembryonic antigen CEA. In addition, radiographic examinations such as a liver contrast-enhanced CT or MRI, as well as a chest CT and/or bone scan, were done every 6-9 weeks to assess CAA therapy response. The Modified Response Evaluation Criteria in Solid Tumors were used to assess the tumor response (mRECIST). This report’s most recent follow-up visit occurred on December 2021. The primary outcomes were overall death and CCA recurrence or rejection. The total survival was calculated from the commencement of first chemotherapy cycle until death from any cause or the last visit. Patients who were lost to follow-up were monitored on the last date they were known to be alive, and those who survived were censored on March 2022, the data cutoff date.

### Gemcitabine Plus Cisplatin as Neo-Adjuvant Treatment

Gemcitabine plus Cisplatin regimen consisted of gemcitabine 1000 mg/m2 intravenously (IV) over 30 minutes followed by cisplatin 25 mg/m2 IV over 60 minutes on days 1 and 8 of a 21-day cycle.

### Non- Gemcitabine and Cisplatin as Neo-Adjuvant Treatment

Gemcitabine monotherapy was administered as 1000 mg/m2 intravenously (IV) over 30 minutes on day 1 of a 7-day cycle. Capecitabine was administered as 1250 mg/m2 by mouth twice daily on days 1-14 of a 21-day cycle. The FOLFIRI regimen administered consisted of irinotecan 180 mg/m2 IV over 90 minutes concurrent with leucovorin 400 mg/m2 followed by fluorouracil 400mg/m2 IV push (bolus), then fluorouracil 2400 mg/m2 continuous IV infusion over 46 hours starting on Day 1 of every 14-day cycle, in combination with FOLFIRI.

### Statistical Analysis

For categorical variables, such as demographic and clinical data, as well as CCA recurrence after transplantation, frequencies and proportions were recorded, whereas continuous variables were presented as median and interquartile range or mean (standard deviation [SD]). For categorical variables, the Chi-square or Fisher’s exact tests were used, while for continuous variables, the Kruskal-Wallis test or unpaired t-test was used. The Kaplan-Meier curves were calculated in strata defined by Gemcitabine/Cisplatin groups for overall survival and CCA-free survival. The log-rank test was used to compare differences between groups. Stata version 17.0 was used for all analyses (StataCorp LLC, College Station, TX, USA). A p-value of 0.05 was considered to be statistically significant (25, 26).

## Results

**Figure 1:**
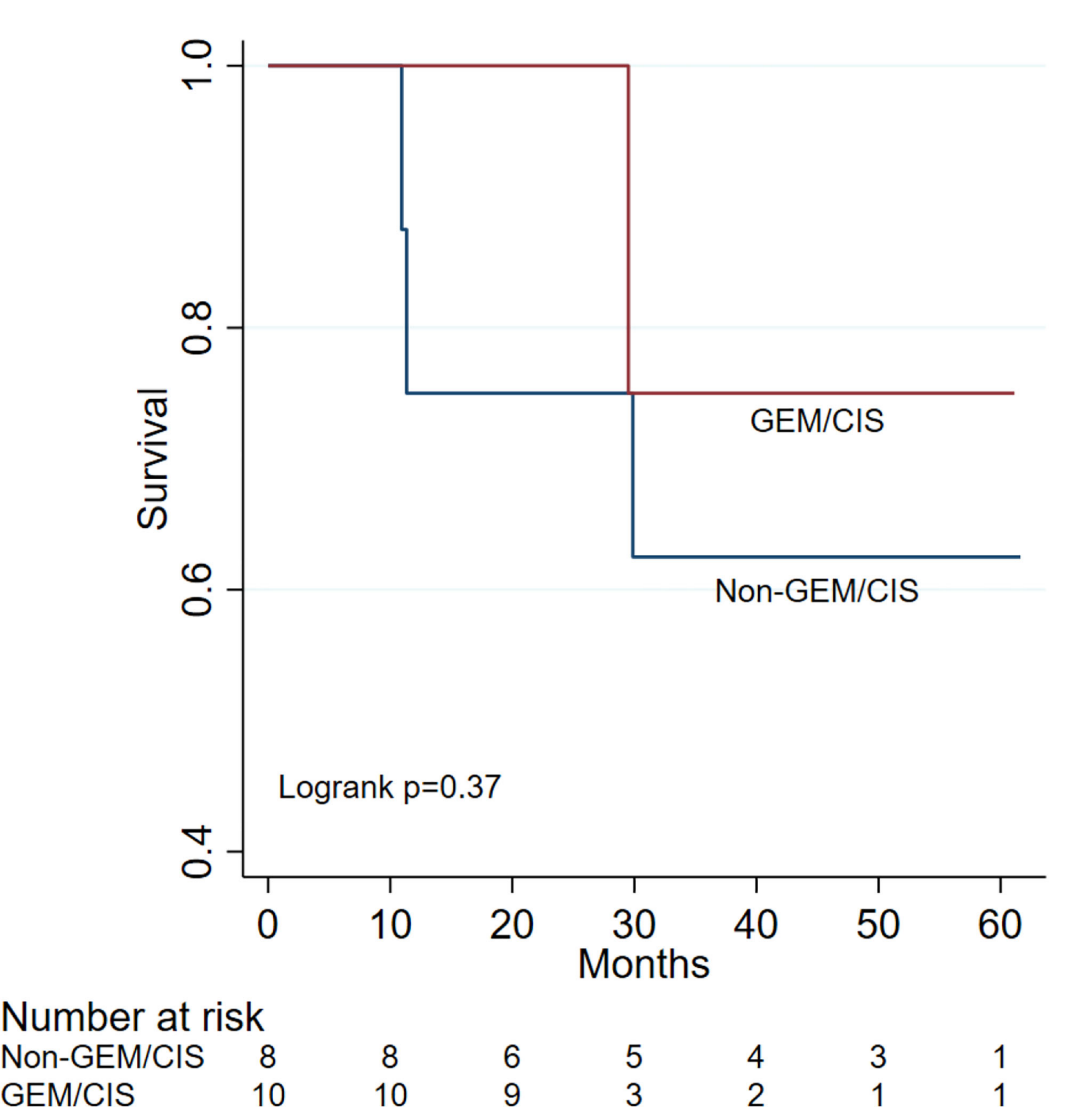
Gemcitabine Plus Cisplatin Versus Non- Gemcitabine and Cisplatin as Neo-adjuvant for Treatment Cholangiocarcinoma Patients Prior to Liver Transplantation, the overall survival was 75% (95% CI 31-93%) at both years 1 and 2; 63% (95% CI 23-86%) at years 3 to 5 in the non-Gemcitabine/Cisplatin group, but in the Gemcitabine/Cisplatin group, the overall survival was 100% (95% CI 100-100%) at both years 1 and 2; 75% (95% CI 13-96%) at both years 3 to 5.

Among 707 patients who underwent liver transplantation, a total of 18 patients (11 males and 7 females) with median age of 61.83 years [interquartile range: 58.27-68.74] had a confirmed diagnosis of cholangiocarcinoma and met the inclusion criteria (**Tables 1**–**3**). Of these 18 patients enrolled, 10 received Gemcitabine/Cisplatin, while 8 patients received either Capecitabine or FOLFIRI. Median months of therapy for patients who received a combination of neoadjuvant Gemcitabine plus Cisplatin was 6.02 months (IRQ: 4-8.33) versus 5.92 months (IRQ: 2.62-10.85) in the non-Gemcitabine/Cisplatin group (p-value=0.93). One patient (10%) in the Gemcitabine/Cisplatin group and 3 patients (37.5%) in the non-Gemcitabine/Cisplatin group had reported recurrence or metastasis (p-value=0.27). Months for recurrence or metastasis after transplantation was 20.1 (IRQ: 20.1-20.1) in the Gemcitabine/Cisplatin group and 9.5 (8.9-12.47) months for the non-Gemcitabine/Cisplatin group (p-value=0.18). All patients received chemotherapy in the neo-adjuvant setting while awaiting liver transplantation. Median months of follow-up in the Gemcitabine/Cisplatin group was 28.35 (27.1-32.23), versus 40.12 (20.6-56.22) months in the non-Gemcitabine/Cisplatin patients (p-value=0.25). The survival differences between non-Gemcitabine/Cisplatin group and Gemcitabine/Cisplatin group showed in **Figure 1** (log rank p=0.37). In the non-Gemcitabine/Cisplatin group, overall survival was 75% (95% CI 31-93%) at both years 1 and 2; 63% (95% CI 23-86%) at years 3 to 5. In the Gemcitabine/Cisplatin group, overall survival was 100% (95% CI 100-100%) at both years 1 and 2; 75% (95% CI 13-96%) at both years 3 to 5. Three patients in the non-Gemcitabine/Cisplatin group died at 328 days, 340 days, and 896 days, respectively. One patient in the Gemcitabine/Cisplatin group died at 885 days. No adverse events were reported after liver transplantation including the patients who had confirmed recurrent disease.

**Table 1:** Transplant related outcomes in patients who received Gemcitabine plus Cisplatin as neo-adjuvant treatment for cholangiocarcinoma prior to liver transplantation.

Patients ID	Sex	Native Liver Diagnosis	Treatment	Treatment Duration- Days	Days to Transplant	Recurrence or Rejection	Days to The Date of Recurrence or Rejection	Days to The Last Follow up	Days to Death
1	Female	Hilar CCA	Gem/Cis	603	8	no		813	
2	Male	CCA	Gem/Cis	149	5	Yes	603	871	885
3	Male	CCA	Gem/Cis	250	20	no		824	
4	Male	CCA	Gem/Cis	120	369	no		967	
5	Male	CCA	Gem/Cis	83	472	no		1405	
6	Male	Hilar CCA	Gem/Cis	161	64	no		418	
7	Male	Hilar CCA	Gem/Cis	201	5	no		812	
8	Male	CCA	Gem/Cis	206	79	no		831	
9	Male	IHCCA	Gem/Cis	77	445	no		1834	
10	Female	IHCCA	Gem/Cis	200	113	no		870	

**Table 2:** Transplant related outcomes in patients who received Non- Gemcitabine/Cisplatin as neo-adjuvant treatment for cholangiocarcinoma prior to liver transplantation.

Patients ID	Sex	Native Liver Diagnosis	Treatment	Treatment Duration- Days	Days to Transplant	Recurrence or Rejection	Days to Recurrence or Rejection	Days to Last Follow up	Days to Death Date
1	Female	IHCCA	FOLFIRI	166	326	yes	374	896	896
2	Male	IHCCA	Capecitabine	189	0	no		1849	
3	Female	HCCA	Capecitabine	35	8	yes	285	315	328
4	Female	H CCA	Capecitabine	122	6	no		1672	
5	Female	HCCA	Gemcitabine	213	96	no		1701	
6	Female	IHCCA	Capecitabine	438	11	no		1137	
7	Male	IHCCA	Capecitabine	792	7	no		1270	
8	Male	IHCCA	FOLFIRI	26	26	Yes	267	221	366

**Table 3:** Baseline Characteristics of Evaluable Patients.

Variables	Total	Non-GEM/CIS	GEM/CIS	p-value
	N=18	N=8	N=10	
Age	61.83 (58.27-68.74)	56.81 (41.66-65.60)	62.71 (60.02-71.87)	0.11
**Gender**				0.14
Female	7 (38.89)	5 (62.50)	2 (20.00)	
Male	11 (61.11)	3 (37.50)	8 (80.00)	
**Recurrence or Rejection**				0.27
No	14 (77.78)	5 (62.50)	9 (90.00)
Yes	4 (22.22)	3 (37.50)	1 (10.00)
**Recurrent Time (Months)**				0.18
	10.98 (9.2-16.28)	9.5 (8.9-12.47)	20.1 (20.1-20.1)
**Chemotherapy Duration (Months)**				
	5.92 (4-8.33)	5.92 (2.62-10.85)	6.02 (4-8.33)	0.93
**Death**				0.27
No	14 (77.78)	5 (62.50)	9 (90.00)
Yes	4 (22.22)	3 (37.50)	1 (10.00)
**Follow-up Time (Months)**				0.33
	29.68 (27.1-46.83)	40.12 (20.6-56.22)	28.35 (27.1-32.23)

Data are presented as median (interquartile range) for continuous variables, and n (%) for categorical variables. Fisher’s exact test for categorical variables and Mann-Whitney test for continuous variables were used to compare Non-GEM/CIS and GEM/CIS.

## Discussion

IHCC incidence has been increasing in the recent decades, in a rate of up to 14% annual increase in frequency, this is due to expanding prevalence of hepatitis C infection worldwide, in addition to increased rates of non-alcoholic steatohepatitis and obesity-related non-alcoholic fatty liver disease globally. Which are considered as acknowledged risk factors for IHCC. However, the available treatment options to make the most effective outcomes for cholangiocarcinoma patients who require liver transplantation are still debatable.

Currently, gemcitabine and cisplatin combination are considered first line treatment for advanced cholangiocarcinoma based on the randomized, controlled, phase III ABC-02 study. Of note, results from a phase II trial suggested that the addition of nab-paclitaxel to gemcitabine and cisplatin prolonged progression-free and overall survival compared to historical controls who received gemcitabine and cisplatin alone. Although there are no head to head randomized controlled trials to support preference for the other fluorouracil, capecitabine or gemcitabine based regimens, it is noteworthy that unresectable tumors in 20% of patients became resectable after treatment with gemcitabine, cisplatin, and albumin-bound paclitaxel. In the second line setting, treatment with FOLFOX (in patients who had previously received Cisplatin and gemcitabine) is the preferred therapy based on improved overall survival compared to active symptom control alone in the randomized Phase II ABC-06 study.

Regarding IHCCA management, Fluorouracil- or capecitabine-based regimens combined with oxaliplatin and gemcitabine, or gemcitabine and cisplatin are recommended treatment options in the neoadjuvant/adjuvant setting. Of note, beyond the Mayo clinic criteria which demonstrated long-term survival using neoadjuvant chemoradiotherapy followed by LT in selected patients with unresectable CCA, other pivotal trials that have examined this strategy have also highlighted the importance of rigorous patient selection for optimal outcomes. Rea and colleagues (27) compared survival outcomes after neoadjuvant chemoradiation and liver transplant versus survival after resection for patients with hilar cholangiocarcinoma. Results were in favor of liver transplantation with neoadjuvant chemoradiation as that strategy achieved better survival compared to resection (one-, 3-, and 5-year patient survival were 92%, 82%, and 82% after transplantation versus 82%, 48%, and 21% after resection (P = 0.022).

There was also less recurrence in the group who received neoadjuvant chemoradiation prior to transplant than the conventional resection group (13% vs 27%). In addition, Darwish and colleague’s (19) analysis of data from multiple transplant centers that employed neoadjuvant chemoradiation and liver transplantation for unresectable perihilar cholangiocarcinoma reported a 65% rate of recurrence-free survival after 5 years. However, there was an 11.5% drop-out rate after 3.5 months of therapy which emphasized the appropriateness of the United Network of Organ Sharing standardized model of end-stage liver disease (MELD) exception for this disease.

Furthermore, out of 27 patients with unresectable cholangiocarcinoma, Diugnan et al. (28) reported long-term overall survival of 94% and 61% at 1 and 4 years in 16 patients who received neoadjuvant chemoradiation prior to transplant. Despite the fact that only patients with progression-free events were selected, short term mortality was reported to be high and patient selection and refinement of neoadjuvant regimens were identified as an area for further exploration. Although survival outcomes with neoadjuvant chemoradiation prior to liver transplant showed promise and reduced overall tumor burden, drop-out rates prior to transplant, poor outcomes in patients with extrahepatic disease, and high short-term mortality were some notable clinically significant outcomes highlighted in these pivotal studies described above.

For patients with unresectable intrahepatic cholangiocarcinoma, radiation therapy may be utilized for locoregional treatment. Evidence for this is limited to non-randomized trials which showed improved 3-year overall survival and 3-year local control when higher doses of radiation were utilized compared to lower doses. In another study, improved 2-year overall survival and progression free survival rate were observed when hypofractionated proton therapy was utilized for patients with unresectable intrahepatic cholangiocarcinoma (29–31).

However, from an outcome perspective, our institution’s experience with radiation prior to transplant has been marginal and complications due to scarring from radiotherapy could potentially delay transplantation. Consequently, for this study, we elected to investigate the benefits of neoadjuvant chemotherapy without radiation in cholangiocarcinoma patients prior to transplant as a novel strategy and elucidate the optimal regimen based on our institution’s experience. In the present study the overall survival in the Gemcitabine plus Cisplatin group 100% (95% CI 100-100%) at both years 1 and 2, was notably longer than the non-Gemcitabine/Cisplatin group, [75% (95% CI 31-93%)]. Moreover, the overall survival rate of 3 and 5 years were longer in the Gemcitabine plus Cisplatin group 75% [95% CI 13-96%] at years 3 to 5 versus the non-Gemcitabine/Cisplatin group 63% [95% CI 23-86%] at years 3 to 5. No adverse effects were observed after liver transplantation. From a mortality perspective, three patients in the non-Gemcitabine/Cisplatin group died at 328 days, 340 days, and 896 days, respectively (**Table 3**). However, one patient died in the Gemcitabine/Cisplatin group at 885 days. In addition, the recurrence rate or metastasis reported in the present study for patients treated with Gemcitabine pus Cisplatin was found to be 10% (one patient), compared to 3 patients (37.5%) in the non-Gemcitabine/Cisplatin group. It is worth highlighting that all patients in the neo adjuvant setting received chemotherapy while awaiting liver transplantation. The time to recurrence or metastasis post liver transplantation in this study was significantly longer in the Gemcitabine plus Cisplatin group [20.1 (IRQ: 20.1-20.1) months] versus the non-Gemcitabine/Cisplatin group [9.5 (8.9-12.47) months] (p-value=0.18). Median months of therapy in patients who received a combination of neoadjuvant Gemcitabine plus Cisplatin was 6.02 months (IRQ: 4-8.33) versus 5.92 months (IRQ: 2.62-10.85) in the non-Gemcitabine/Cisplatin group (p-value=0.93). In contrast the median months needed for follow up in the non-Gemcitabine/Cisplatin patients (p-value=0.33) was higher at 40.12 (20.6-56.22) days compared with 28.35 (27.1-32.23) months observed in the Gemcitabine/Cisplatin group (**Tables 1**,**2**).

While our institutions’ experience with managing CCA has been modest with rare tumor, we highlight the clinical utility of Gemcitabine plus Cisplatin Versus Non-Gemcitabine and Cisplatin regimens as neo-adjuvant treatment prior to liver transplantation. Some limitations of this study include small sample size, the retrospective nature of the analysis, and the lack of a control arm. A prospective randomized clinical trial may be necessary in the future to demonstrate the superiority of Gemcitabine plus Cisplatin for CCA patients prior to liver transplantation.

## Conclusion

Based on the previously described data, the Gemcitabine plus Cisplatin group demonstrated improvement in outcomes compared to the non-Gemcitabine/Cisplatin group in CCA liver transplanted patients. We recommend further evaluation of neoadjuvant treatment with Gemcitabine plus Cisplatin and no concomitant radiation prior to liver transplantation as a novel strategy for improving outcomes in patients with unresectable cholangiocarcinoma requiring significant coordination of care.

## Data Availability Statement

The original contributions presented in the study are included in the article/Supplementary Material. Further inquiries can be directed to the corresponding author.

## Ethics Statement

The studies involving human participants were reviewed and approved by Houston Methodist Institutional Review Board approved protocol (IRB ID: PRO00032826). Written informed consent for participation was not required for this study in accordance with the national legislation and the institutional requirements.

## Author Contributions

MA, AE, and RG contributed to the conception; MA, HA-R, GU, and AE contributed to abstract writing; AE contributed to literature search and acquisition; AE, AA, DV, and MA are responsible for drafting and revising the manuscript; JX, HA-R, and AE contributed to data analysis and interpretation; MA, JX, GU, AS, RM, SK, AA, DV, HA-R, and AE were responsible for the critical revision of the manuscript for intellectual content; and all authors issued final approval for the version to be submitted. All authors affirm final approval of the version to be published and agree to be accountable for all aspects of the work in ensuring that questions related to the accuracy or integrity of any part of the work are appropriately investigated and resolved.

## Conflict of Interest

The authors declare that the research was conducted in the absence of any commercial or financial relationships that could be construed as a potential conflict of interest.

## Publisher’s Note

All claims expressed in this article are solely those of the authors and do not necessarily represent those of their affiliated organizations, or those of the publisher, the editors and the reviewers. Any product that may be evaluated in this article, or claim that may be made by its manufacturer, is not guaranteed or endorsed by the publisher.
